# Fabrication of Stretchable Circuits on Polydimethylsiloxane (PDMS) Pre-Stretched Substrates by Inkjet Printing Silver Nanoparticles

**DOI:** 10.3390/ma11122377

**Published:** 2018-11-26

**Authors:** Jumana M. Abu-Khalaf, Loiy Al-Ghussain, Ala’aldeen Al-Halhouli

**Affiliations:** Mechatronics Engineering Department/NanoLab, School of Applied Technical Sciences, German Jordanian University, P.O. Box 35247, Amman 11180, Jordan; loui.essam@hotmail.com (L.A.-G.); alaaldeen.alhalhouli@gju.edu.jo (A.A.-H.)

**Keywords:** stretchable circuits, pre-stretching, PDMS, inkjet printing, silver nanoparticle ink, sensors platform

## Abstract

Several research methodologies have recently been developed to allow for the patterning of conductive lines on elastomeric rubber substrates. Specifically, various conductive materials, substrates, and fabrication techniques were investigated to develop stretchable circuits. One promising technique recommends the application of axial strain on an elastomer substrate prior to patterning conductive lines on it. When the substrate is released, conductive lines buckle to form waves, making the circuit stretchable. However, the majority of applications of stretchable circuits require fitting them to two-dimensional surfaces, such as the human body. Hence, in this paper we propose the concept of radial pre-stretching of the substrates to enhance the stretchability of the fabricated circuits. In particular, straight silver conductive lines were deposited on a polydimethylsiloxane (PDMS) surface using inkjet printing technology, and subsequently tested under both axial and radial loads. Radial pre-stretching was compared to axial pre-stretching, resulting in an improved performance under radial loads. The optimal performance was achieved by pre-stretching the PDMS substrate with a radial strain of 27%. This resulted in stretchable circuits which could sustain radial loads with an average breakdown strain of approximately 19%. Additionally, horseshoe patterns were printed on radially pre-stretched PDMS substrates and their performance was compared to that of their straight line counterparts. Though these patterns are generally favorable for the fabrication of stretchable circuits, the optimal horseshoe pattern examined in this study could only sustain up to 16% radial strain on average when radially pre-stretched by 27%.

## 1. Introduction

The development of stretchable circuits presents an intellectually rich set of challenges, to which scientists and researchers attempt to propose novel solutions. This emerging field allows for the fabrication of electronic devices with higher reliability, functionality, and miniaturization. Furthermore, stretchable circuits can sustain mechanical deformations, up to a certain limit, which makes them suitable for several unconventional applications, such as bioelectric monitoring devices, electronic skin applications, and epidermal circuitry [1,2]. In the literature, several experimental techniques can be found for the fabrication of stretchable circuits using different substrates, such as polydimethylsiloxane (PDMS) [2,3,4,5,6], and silicon wafers [1,4]. Additionally, different deposition technologies such as inkjet printing [7,8,9,10], photolithography [6], and screen printing [5,11,12], have been investigated. Moreover, research conducted on liquid metal demonstrates its potential for the fabrication of circuits with high stretchability [3]. Nevertheless, the use of liquid metal results in costly and possibly hazardous circuits, depending on the application. On the other hand, inkjet printing offers an affordable and low-cost fabrication method for stretchable circuits [7].

Traditional lithography techniques have the capability to fabricate stretchable circuits on various elastomeric materials. However, this technology requires expensive and complex fabrication steps which makes it less favorable in the presence of alternative technologies such as inkjet printing [13]. Not only is inkjet printing a low-cost technique, but it also requires less complex fabrication steps and reduces waste material in the fabrication process. Inkjet printing has two modes of operation; drop on demand (DoD), and continuous inkjet printing (CIJ). In the DoD mode of operation, printers are characterized by their high accuracy in the placement of the ink droplets with efficient control and no wasted ink. The nozzles of the inkjet printer usually contain piezoelectric actuators, which each release a single ink drop when induced by a digital signal or waveform; whereas, the CIJ mode releases a continuous fluid stream via the vibration of a piezoelectric actuator. The latter mode requires larger amounts of ink, thus the DoD mode presents a more advantageous option [14].

**Figure 1:**
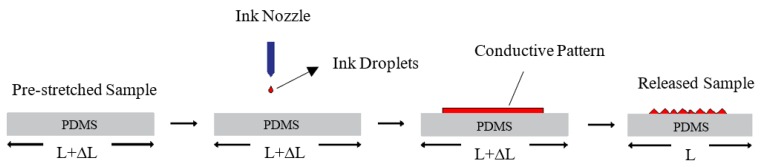
Schematic diagram of the axial pre-stretching technique adapted from Reference [18].

Several fabrication methods have recently been developed to fabricate stretchable circuits, which maintain electrical conductivity when stretched, on elastomeric rubber substrates. Some of the most promising techniques include: depositing in-plane conductive tortuous patterns, such as horseshoe and sinusoidal patterns, on stretchable substrates [15,16]; depositing conductive patterns on wavy surfaces which do not induce significant strains [17]; and depositing conductive patterns on axially pre-stretched substrates which will form out-of-plane wavy patterns after release [18]. Figure 1 shows a schematic diagram of the axial pre-stretching technique.

Stretchable circuits are usually implemented in wearable sensors which are required to conform to 2D curvatures, such as the human body [8]. Hence, pre-stretching the substrate axially is not sufficient to achieve the required stretchability. In this paper, we build on the pre-stretching technique by radially pre-stretching PDMS substrates prior to inkjet printing silver nanoparticle (NP) based ink on them. Although several research studies examine the effect of axial pre-stretching on enhancing the stretchability of fabricated circuits [18,19,20,21,22], the concept of radial pre-stretching has hardly been investigated in the literature [8]. Moreover, the stretchability achieved by printing conductive tortuous patterns on top of radially pre-stretched PDMS substrates, is examined in this study. Accordingly, test circuits are composed of straight and horseshoe silver patterns printed on top of PDMS substrates. Finally, the stretchability of axially and radially pre-stretched samples are compared under both axial and radial loads.

## 2. Materials and Equipment

In this section, the materials and equipment required for the fabrication of stretchable circuits using inkjet printing are introduced.

### 2.1. Elastomer Substrates

Polydimethylsiloxane (PDMS) is one of the most commonly used silicon-based elastomers for the fabrication of stretchable circuits. PDMS is durable, commercially available, compatible with biomedical applications, and safe for use with the human body [18,23,24,25]. In this study, Dow Corning^®^ Sylgard 184 (The Dow Chemical Company, Wiesbaden, Germany) was used for the fabrication of PDMS substrates, where the transparent PDMS was formed by mixing a base material and a curing agent with a ratio of 10:1, respectively. The PDMS mixture was poured into rectangular (100 mm × 40 mm) and circular (86 mm diameter) acrylic molds (Perspex, Watford, UK), producing substrates with thicknesses of 1 mm. Subsequently, the molds were placed into a vacuum oven (MMM Group, Munich, Germany) for degasification at room temperature for 30 min. Finally, the PDMS mixture was cured by placing it in the oven at 70 °C for 2 h [13,26].

**Figure 2:**
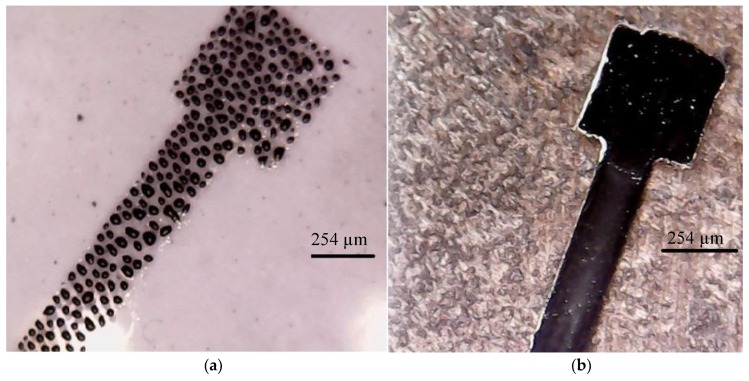
Printed silver NP ink on: (**a**) untreated PDMS surface; and (**b**) treated PDMS surface.

PDMS has a hydrophobic nature which means that the formation of conductive patterns on its surface is not possible without proper treatment. The size and the stability of the printed lines are also affected by the wettability of the substrate [13,26]. Therefore, it is essential to treat the PDMS surface, either chemically or physically, prior to inkjet printing of NP ink to enhance the hydrophilicity of the PDMS surface [13,26]. In this study, plasma barrel etching technology was used for the treatment of PDMS surfaces. Specifically, a PDMS sample was placed in the ZEPTO Diener plasma etcher (Diener electronic GmbH, Ebhausen, Germany) for 15 min at full power (50 Watt). Figure 2 illustrates the effect of plasma etching on the hydrophilicity of the PDMS surface and the quality of the printed silver conductive lines on untreated and treated surfaces, respectively.

### 2.2. Inkjet Printing

A Fujifilm Dimatix Material Printer DMP-2800 (Fujifilm, New Hampshire, USA) was used to deposit conductive ink on PDMS samples. This printer uses the DoD mode of operation, where it has nozzles with piezoelectric technology that ensure accurate deposition of silver NPs in a cost-effective way. Moreover, electronic pulses sent to the piezoelectric nozzles can be manipulated in order to optimize the printing parameters, where a drop-watch camera is provided to ease this procedure. Cartridges, which contain a 1.5 mL reservoir filled with silver ink, are used to reduce the waste in ink. These cartridges can be easily replaced to facilitate the printing of different fluids. Each cartridge has 16 nozzles that are linearly spaced at 254 microns with typical droplet sizes of 1 and 10 picolitres.

The desired pattern is fed to the printer via a pattern editor program with a specific resolution to meet the required drop spacing (DS). The resolution of the pattern can by calculated using Equation (1) as reported in Reference [16], where the optimal DS for NP silver ink is found to be 30 µm.

(1) Rs=25400/DS

where Rs is the resolution of the pattern (dpi) and DS is the drop spacing (µm). In this study, all conductive lines were printed with the optimal DS of 30µm.

Silverjet DGP-40LT-15C from Sigma Aldrich (Sigma-Aldrich, Darmstadt, Germany) was chosen as the conductive ink used in the experiments conducted in this study. It consists of a solvent (30–35 weights % dispersion in triethylene glycol monomethyl ether) with suspended silver nanoparticles. This ink is characterized by its high electrical conductivity, low chemical reactivity, and chemical stability [27]. The sliver NP ink has a viscosity of 10–18 cP and a surface tension of 35–40 dyn/cm which makes it suitable for use with the Dimatix inkjet printer.

## 3. Methodology

In this section, the methodology for fabricating reliable stretchable circuits is introduced.

### 3.1. Conductive Patterns

In order to examine the effect of pre-stretching on the stretchability of the fabricated circuits, the simplest circuit was chosen—a straight line with two square pads at its ends. The Dimatix inkjet printer was used to print straight conductive lines, where two layers of ink are deposited to reduce the resistance of the conductive line. The printed silver conductive lines have a thickness in the range of 1200–1800 nm [16,28].

In the literature, it is reported that tortuous patterns increase the stretchability of the fabricated circuits. The work presented in Reference [15] concluded that constructing a horseshoe pattern using lithography could increase the stretchability of the circuit by up to 54%. Inkjet printing was also used to print a horseshoe pattern on PDMS that is capable of withstanding an axial strain of 25% with a resistance under 800 Ω [16]. Therefore, in this study the stretchability of horseshoe circuits was further inspected by applying radial loads to them. Additionally, horseshoe patterns were printed on radially pre-stretched PDMS substrates; in order to analyze the effect of the radial pre-stretching technique on the stretchability of the circuit.

**Figure 3:**
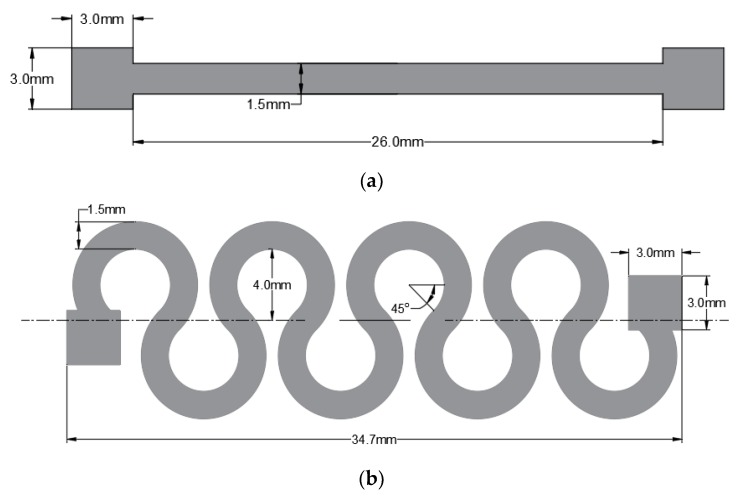
The shape and the dimensions of the examined patterns: (**a**) straight line, and (**b**) horseshoe.

Based on the results presented in [16], the optimal horseshoe pattern has 4 cycles, a width of 1.5 mm, and an amplitude of 4 mm. Figure 3 illustrates the shape and the dimensions of the straight line and horseshoe patterns examined in this study. Two layers of the horseshoe pattern were printed to match the thickness of the straight line and conduct an unbiased comparison. In Reference [16] it is also reported that increasing the width of the pattern increases the stretchability of the circuit. Therefore, an additional horseshoe pattern with a linewidth of 2 mm, 4 cycles, an amplitude of 4 mm, and a thickness of 2 layers was printed and tested.

### 3.2. Pre-Stretching of PDMS Samples

A PDMS sample is pre-stretched either axially or radially using in-lab developed setups. For axial pre-stretching, a vice with magnetic clamps is used to stretch rectangular and circular PDMS substrates. The amount of pre-stretching strain is measured manually using a caliper. The axially pre-stretched rectangular substrates are tested under axial load, while the pre-stretched circular substrates are tested under radial load.

In addition, a radial stretcher with an Arduino microcontroller, which was replicated from the work presented in Reference [29], was used to apply radial loads to the samples. The design was rescaled such that 18 holders were used to stretch a PDMS sample in all directions. A prototype was fabricated from a 6 mm thick Poly methyl methacrylate (PMMA), known also as acrylic, using a laser cutter. This thickness is necessary to withstand expected stresses without fracturing. At the zero position (0% strain), the stretcher had a diameter of 72 mm while at the maximum strain (125%) the stretcher had a diameter of 162 mm.

A circular PDMS substrate was mounted on the radial stretcher via magnetic clamps which were connected to acrylic arms that move as a stepper motor runs, causing the PDMS sample to stretch. While on the stretcher, the sample is placed between a circular metal plate and circular magnetic piece. This maintains the pre-stretching strain after removing the sample from the stretcher. Next, a circular metal clamp covered with wool to reduce the contact stress, was secured around the circular plate. This held the PDMS sample in place and allowed for removing the magnetic piece.

**Figure 4:**
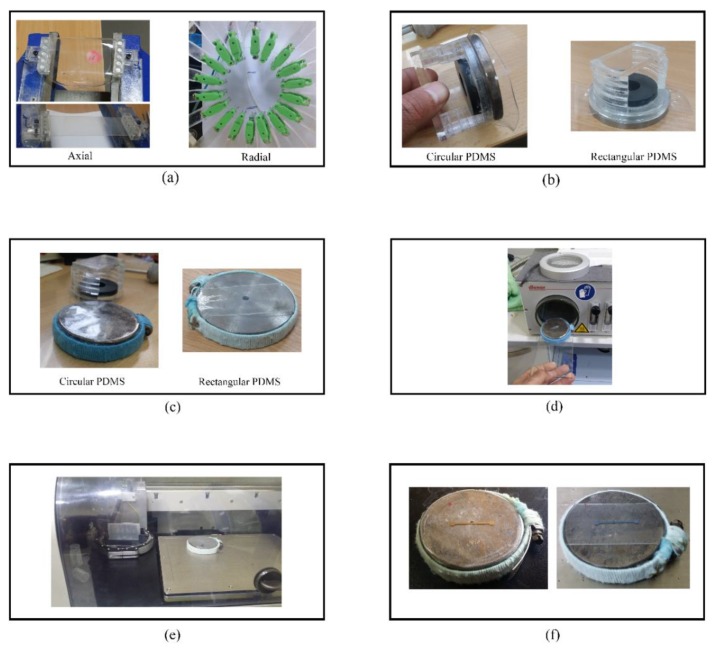
The fabrication process of the stretchable circuits. (**a**) Axial and radial pre-stretching of PDMS; (**b**) placing the stretched PDMS sample between a metal plate and magnetic piece to maintain the pre-stretching strain; (**c**) securing the PDMS sample on the plate using the circular clamp; (**d**) surface treatment of the PDMS sample; (**e**) printing conductive lines using the inkjet printer; and (**f**) sintering the ink in the oven.

Subsequently, the clamped PDMS sample was placed into the plasma etcher for PDMS surface treatment. Then, the PDMS sample was placed into the inkjet printer to print conductive sliver patterns on its surface. Next for ink sintering, the circuit was placed in an oven at 150 °C for one hour. Finally, the circular clamp was removed and the circuit was again mounted on the radial stretcher for testing. Figure 4 demonstrates the proposed fabrication process of the stretchable circuits.

### 3.3. Tests Performed

In order to investigate the stretchability of the inkjet-printed circuits, various rectangular and circular PDMS substrates with conductive straight lines were examined. Rectangular substrates were subjected to axial pre-stretching at various strain values, and tested only under axial loads after printing straight conductive lines on them. This test was conducted to establish a reference performance of the axial pre-stretching technique, shown in Figure 1, to which the radial pre-stretching technique proposed in this paper would be compared.

In addition, the circular PDMS substrates were divided into two batches; one batch was axially pre-stretched, and the other was radially pre-stretched. After printing, the first batch was only subjected to radial loads, while the second batch was subjected to both axial and radial loads. The results of the first batch were used as an indication of the performance of axially pre-stretched circuits under radial loads, i.e., 2D curvatures, to demonstrate their disadvantage in such applications. Finally, the results of the second batch were used to demonstrate the effectiveness of radial pre-stretching in enhancing the stretchability of the circuits.

**Figure 5:**
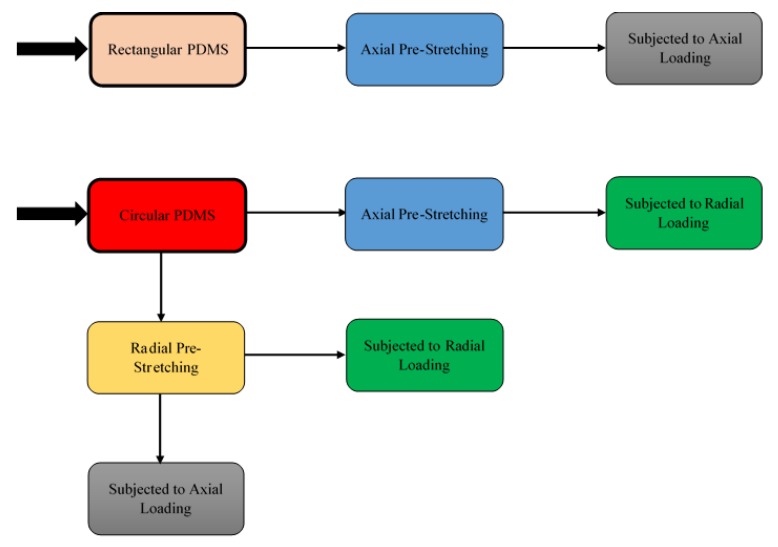
The procedure followed to examine the effect of axial and radial pre-stretching on the stretchability of the printed circuits under axial and radial loads.

Figure 5 illustrates the procedure followed for examining the effects of axial and radial pre-stretching on the stretchability of inkjet-printed circuits under axial and radial loads. It should be noted that the axial and radial loads in all tests were applied with strain steps of 1%. Axial loads were applied using the stretcher by mounting the circuits on two opposite arms vertically. Radial loads were applied by mounting circular circuits on the stretcher via all 18 arms.

Furthermore, cyclic tests were performed under radial loads in order to test the durability of the fabricated circuits. In particular, pre-stretched circuits with both straight lines and horseshoe patterns were tested. It should be noted that normalized resistances are used when plotting the results to eliminate the effect of variations in the initial resistances of the circuits. A normalized resistance ($R_{n}$) can be calculated using Equation (2) as follows:

(2) $$R_{n}=(R_{f}-R_{i})/R_{i}$$

where $R_{f}$ is the final resistance (Ohm) and $R_{i}$ is the initial resistance (Ohm).

## 4. Results and Discussion

### 4.1. Straight Conductive Lines

**Figure 6:**
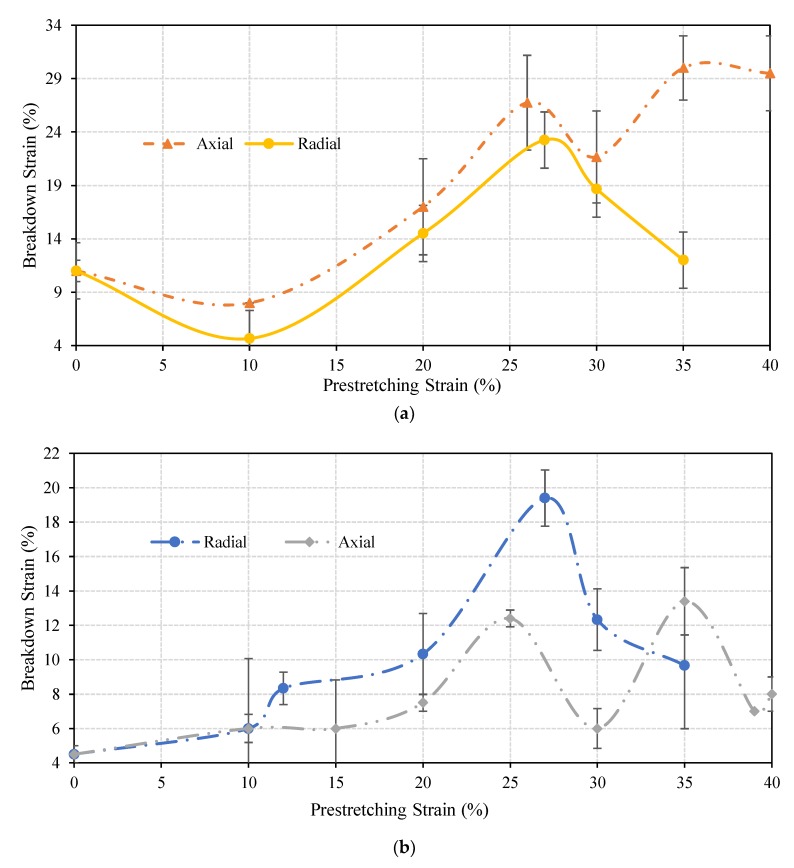
Average breakdown strains of conductive straight lines which were axially and radially pre-stretched with various values and examined under: (**a**) axial load, and (**b**) radial load.

Several axially and radially pre-stretched circuits were tested under axial and radial loads, and the breakdown strain for each circuit was recorded. This is the strain at which the electrical resistance of the circuit exceeded 1 MOhm. Figure 6 illustrates the relationship between the breakdown strain and the amount of the pre-stretching strain under axial and radial loads, respectively.

It can be noticed in Figure 6a that the axially pre-stretched circuits exhibit a slightly better performance under axial loads when compared to radially pre-stretched circuits. However, as shown in Figure 6b, the radially pre-stretched circuits exhibit a much better performance under radial loads. Specifically, a radial pre-stretching strain of 27% results in a maximum average breakdown strain of approximately 19%. On the other hand, a maximum average breakdown strain of approximately 13% is achieved by an axial pre-stretching strain of 35%. The relationship between the pre-stretching strain and the breakdown strain is more or less linear, up to a pre-stretching strain of 27%, whereas beyond this point nonlinearities start to occur.

**Figure 7:**
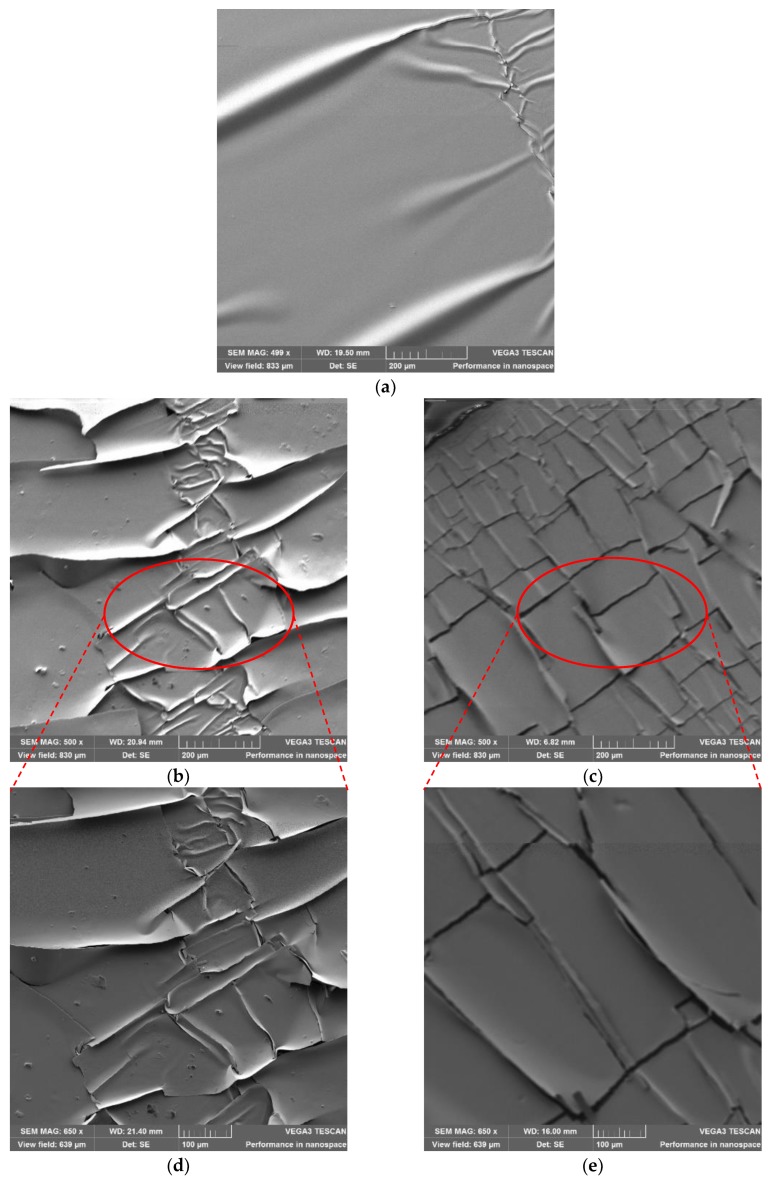
SEM images of straight line patterns at different pre-stretching strains: (**a**) 0%; (**b**) 27% axial pre-stretching with a scale of 200 µm; (**c**) 27% radial pre-stretching with a scale of 200 µm; (**d**) 27% axial pre-stretching with a scale of 100 µm; and (**e**) 27% radial pre-stretching with a scale of 100 µm.

Scanning electron microscopy (SEM), using TESCAN VEGA3 (TESCAN, Brno, Czech Republic), was used to analyze the effects of axial and radial pre-stretching on the topography of the printed silver patterns. Figure 7 shows SEM images of inkjet-printed silver straight lines at 0% and 27% axial and radial pre-stretching strains. The images demonstrate that both pre-stretching methods result in out-of-plane deformations (Figure 7b,c compared to Figure 7a). These deformations enhance the ability of the lines to maintain conductivity under large strains. It is also evident from Figure 7c–f that radial pre-stretching increases the out-of-plane deformation compared to axial pre-stretching, which explains the ability of the radially pre-stretched circuits to sustain higher radial loads.

As aforementioned, most of the applications of stretchable circuits require fitting the circuits to a 2D curvature, such as the human body, which is analogous to the radial loading in this study. It is also reported in the literature that electronics placed at different locations on the human body are usually subjected to deformations up to a strain of 20% [30,31]. Hence, the radial pre-stretching technique presented in this paper demonstrates substantial potential in enhancing the stretchability of wearable circuits.

**Figure 8:**
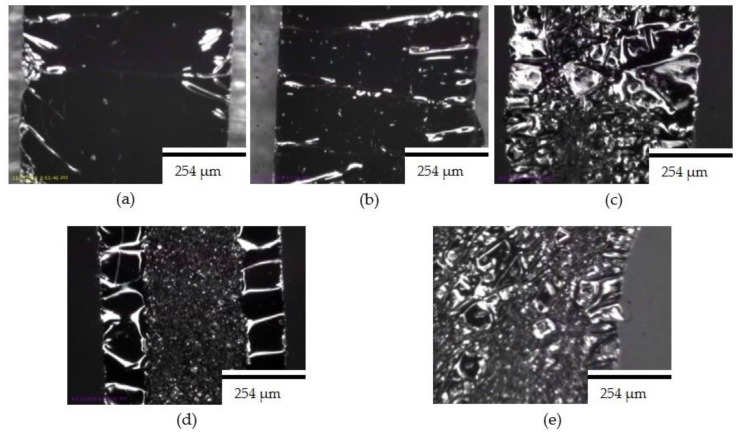
Microscopic images of conductive silver straight lines at various radial pre-stretching values: (**a**) 0%; (**b**) 10%; (**c**) 27%; (**d**) 30%; and (**e**) 35%.

Clearly, radial pre-stretching forms an out-of-plan deformation of the silver ink, which can be restored up to a certain limit when the samples are unloaded. The level of the deformation depends on the amount of pre-stretching strain. However, the relationship is only considered linear up to 27% pre-stretching strain as aforementioned. Figure 8 shows microscopic images of radially pre-stretched circuits at various pre-stretching strains.

**Figure 9:**
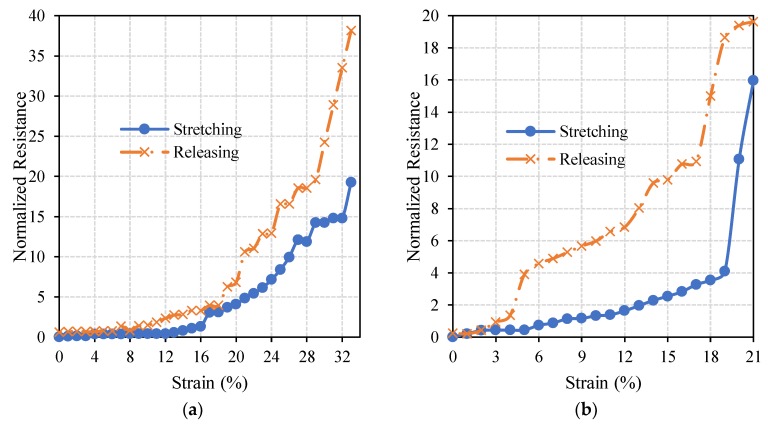
The change in the normalized resistance of the straight line printed on a radially pre-stretched circuit by 27% under (**a**) axial, and (**b,**) radial loads.

**Figure 10:**
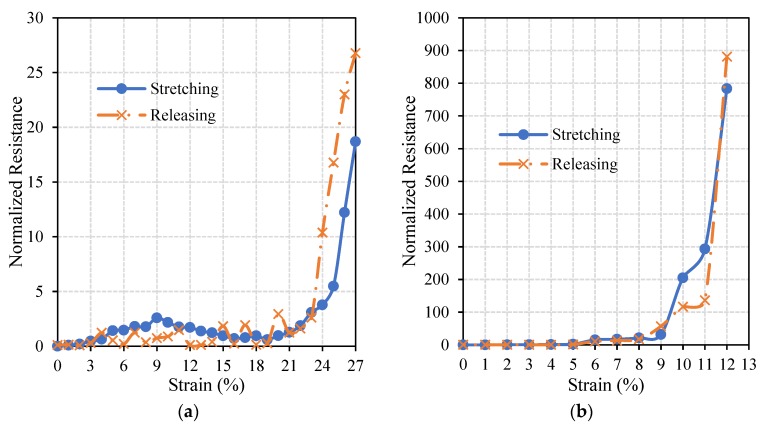
The change in the normalized resistance of the straight line printed on an axially pre-stretched circuit by 35% under (**a**) axial, and (**b**), radial loads.

Circuits which resulted in optimal performances (27% radially pre-stretched and 35% axially pre-stretched circuits) were further examined to inspect their electrical stability under axial and radial loads, as this is one of the crucial characteristics of stretchable circuits. Figure 9 and Figure 10 show normalized resistances of the straight lines printed on the radially and axially pre-stretched optimal circuits, respectively, under axial and radial loads.

It can be noticed in Figure 9a that the resistance of the radially pre-stretched optimal circuit is approximately constant under axial stretching (loading) and releasing (unloading) up to approximately 10% strain. At no load, the measured initial resistance is 32.8 Ohm and it increases up to 664 Ohm as the circuit is gradually loaded to 33% strain. Upon release of the circuit, i.e., gradually unloading the circuit until a strain of 0% is reached, the measured resistance is found to be slightly higher at 52.5 Ohm. In Figure 9b, the resistance of the circuit under radial load has an approximately constant resistance up to 4% strain. However, as the circuit is released (unloaded) the measured resistances significantly diverge from the loading resistance curve. The measured initial resistance is 25.5 Ohm and it increases up to 432.7 Ohm at the maximum strain, then yields to 31.7 Ohm upon unloading.

Figure 10 shows that the resistance of the axially pre-stretched optimal circuit is approximately constant up to 8% strain under radial loads. During axial loading, the measured initial resistance of the circuit is 18 Ohm and it reaches up to 354 Ohm at the breakdown strain. After releasing it decreases to 20 Ohm. Under radial loading, the measured initial resistance is 10.2 Ohm and it reaches up to 8 kOhm at the breakdown strain. After releasing, the resistance decreases back to 13 Ohm.

In both axial and radial loading of the optimal radially pre-stretched circuit, the normalized resistance did not exceed 40. However, for the optimal axially pre-stretched circuit, the normalized resistance under radial load reached approximately 900 at the breakdown strain. These results confirm that radially pre-stretched circuits can better sustain radial loads than their axially pre-stretched counterparts. Moreover, the results obtained from axial loading of axially pre-stretched circuits in this study are comparable to similar experiments reported in the literature. For instance, 2 layers of a 30 mm long and 1 mm wide silver straight line were printed using inkjet printing techniques on a 33% axially pre-stretched PDMS sample in Reference [20]. The printed circuit sustained an axial load of approximately 20%, where the initial resistance of the line was recorded at 5.09 Ohm and the final resistance was 50.9 Ohm. These results are comparable to the ones we obtain when axially pre-stretching a PDMS sample by 30% strain. Specifically, the average breakdown strain is almost 19%, with an initial resistance of 27.8 Ohm and a maximum the resistance of 75.1 Ohm at the breakdown strain.

### 4.2. Conductive Horseshoe Patterns

Several PDMS samples with horseshoe patterns printed on them, with various numbers of layers and linewidths, were tested under radial loads. The desire was to investigate the effect of combining the pre-stretching methodology introduced here, with the use of tortuous patterns on the stretchability of the printed circuits. PDMS samples were radially pre-stretched at a strain of 27% as this pre-stretching value resulted in an optimal performance when printing straight conductive lines, as found in the previous section.

Table 1 demonstrates the effect of the linewidth, number of layers, and the pre-stretching strain on the stretchability of the circuits with horseshoe patterns. The results are consistent with those reported in literature; that is, increasing the width of the pattern increases the stretchability of the circuit [16]. Without radial pre-stretching, the conductivity of the horseshoe pattern with a linewidth of 1.5 mm (1 and 2 layers) is lost at an average strain of 4.5%, while the horseshoe pattern with a 2 mm linewidth sustains a maximum strain of 9%.

Furthermore, the results in Table 1 show that radial pre-stretching of the horseshoe circuit increases its stretchability by approximately three times for the pattern with 1.5 mm linewidth and almost twice for the pattern with 2 mm linewidth. The number of layers does not significantly affect the stretchability of the radially pre-stretched circuit, where a horseshoe circuit of two layers of silver ink has on average an increased stretchability of only 0.5% when compared to a circuit of one layer.

The performance of the circuits with horseshoe patterns without pre-stretching under radial load is comparable to the performance of the circuits with straight lines without pre-stretching. In particular, when comparing both circuits with a linewidth of 1.5 mm, their conductivity is lost at 4.5% strain on average. The conductivity of the optimal radially pre-stretched circuit with a horseshoe pattern (two layers) is lost at a strain of 13.5% on average, while the straight line circuit at the same pre-stretching value lost its conductivity at 19% strain.

**Figure 11:**
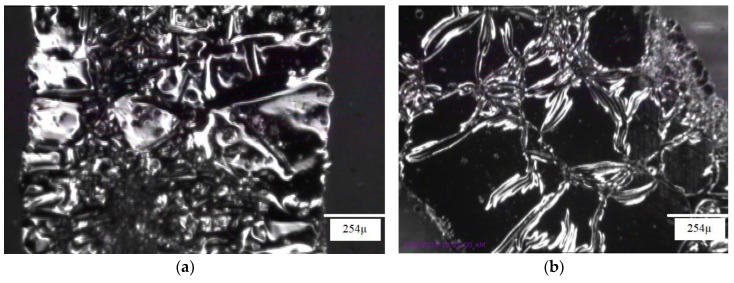
Microscopic images of radially pre-stretched samples at 27% strain with (**a**) straight line, and (**b**) horseshoe patterns.

**Figure 12:**
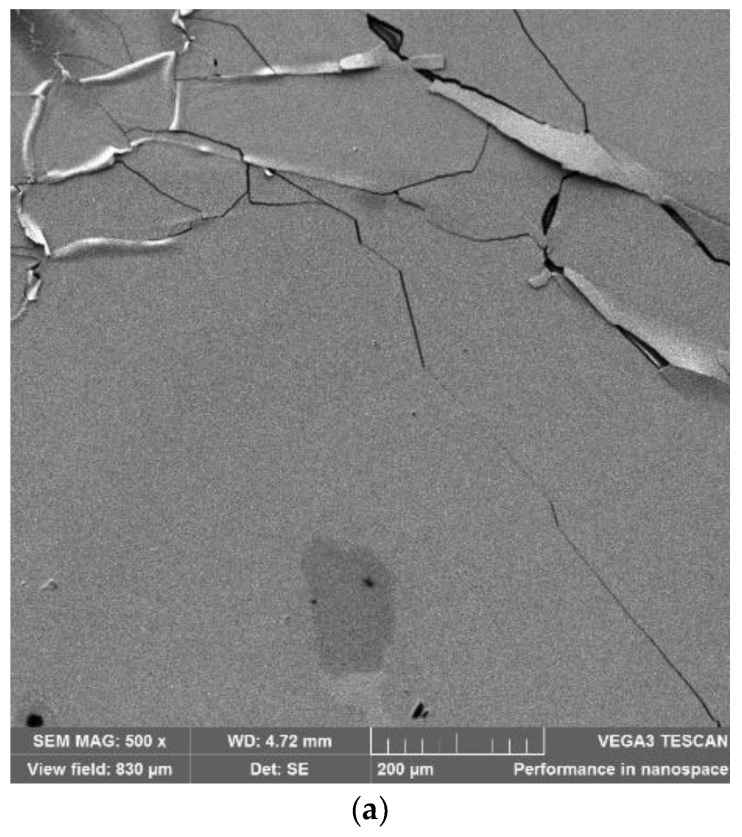

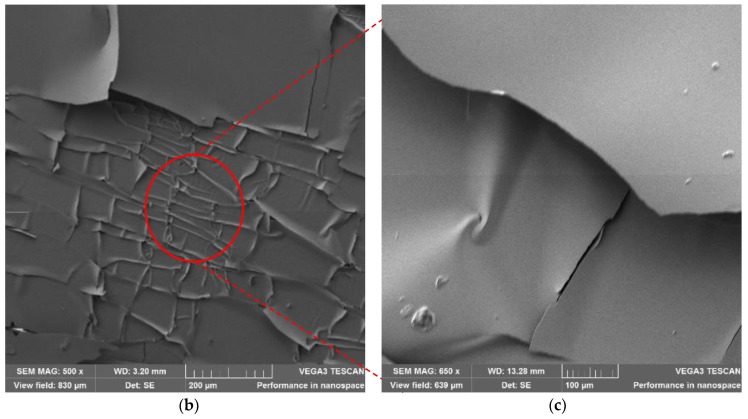
SEM microscopic images of the horseshoe patterns printed on: (**a**) an unstretched sample; (**b**) a 27% radially pre-stretched sample at 200 µm scale; and (**c**) a 27% radially pre-stretched sample at 100 µm scale.

Figure 11 demonstrates microscopic images of samples that were radially pre-stretched with a 27% strain. The printed straight line has fewer cracks than the horseshoe pattern, which might be due to the shape of the horseshoe. This is confirmed by SEM images of horseshoe patterns printed on both unstretched and 27% radially pre-stretched samples, as demonstrated in Figure 12. When comparing these images to those of the straight lines previously shown in Figure 7, one can see that the horseshoe pattern even when unstretched has more cracks, which could explain its deficient performance.

**Figure 13:**
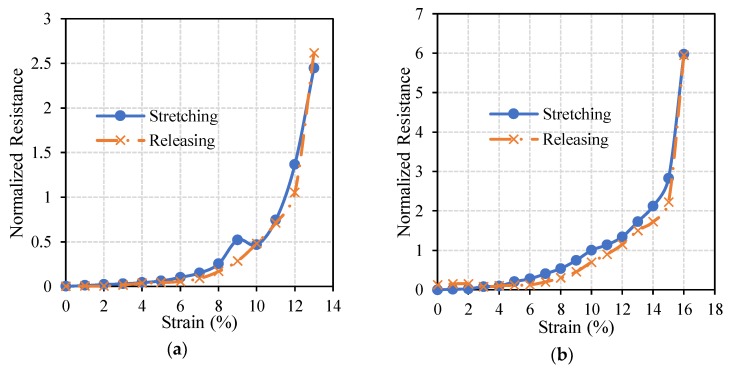

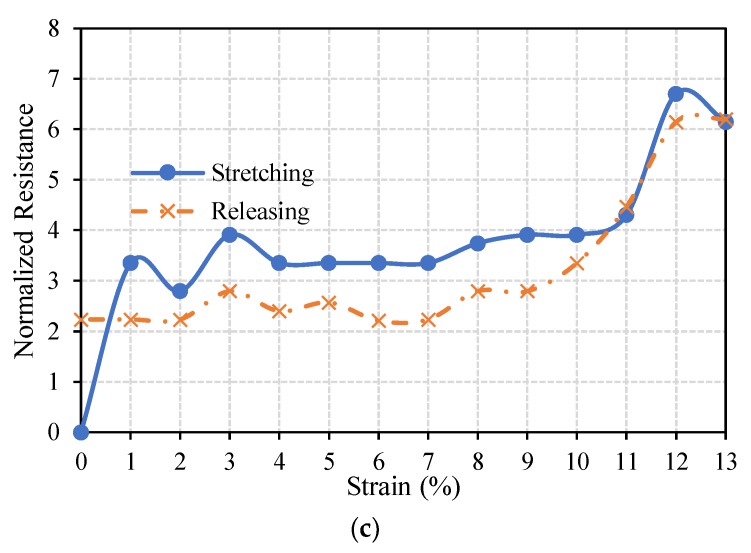
The change in the normalized resistance of the horseshoe pattern with: (**a**) one layer and a linewidth of 1.5 mm; (**b**) one layer and a linewidth of 2 mm; and (**c**) two layers and a linewidth of 1.5 mm, printed on the optimal radially pre-stretched samples under radial load.

The resistance of the horseshoe patterns remains almost constant during radial loading up to almost 8%, beyond which point the resistance starts to increase. Figure 13 shows the normalized resistance of the horseshoe pattern printed on radially pre-stretched samples by 27% strain under radial load. Upon releasing the load applied on the circuit, the resistance almost returns to its original value. The initial resistance of the pattern, with a width of 1.5 mm and a thickness of one layer, is 69.1 Ohm at zero strain, and reaches 698 Ohm at 13% strain. After removing the load, the resistance decreases to 71 Ohm. While for the 2-layer circuit the initial resistance is measured at 37 Ohm and reaches to 84 Ohm at 13% strain. It finally decreases to 57 Ohm after releasing the load. The horseshoe pattern with 2 mm linewidth and one layer has an initial resistance of 40 Ohm at zero strain, which reaches 279 Ohm at 16% strain and decreases to 45 Ohm after removing the load.

### 4.3. Radial Cyclic Test

**Figure 14:**
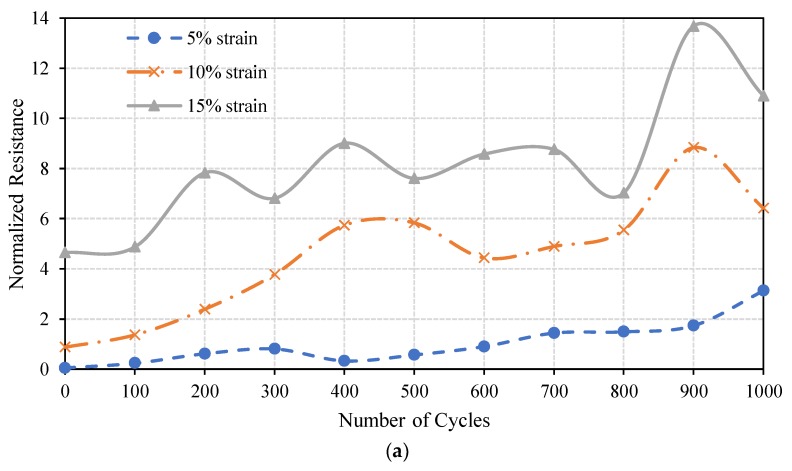

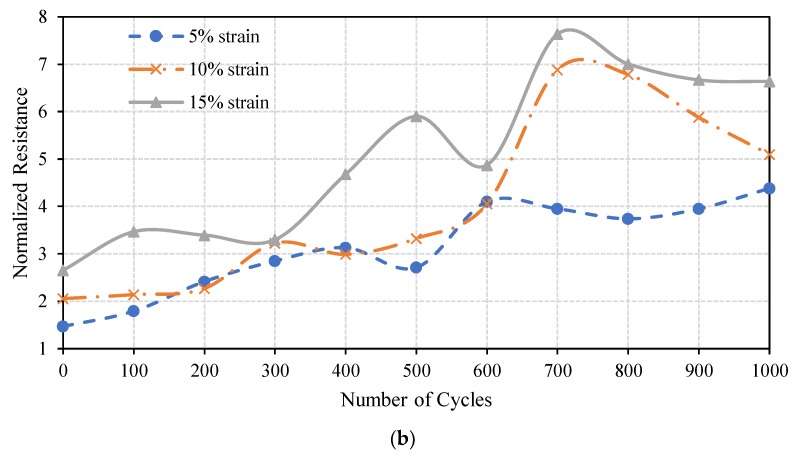
Normalized resistances of (**a**) the straight line, and (**b**) the 1-layer 2 mm horseshoe patterns, printed on the optimal radially pre-stretched sample cycled up to 1000 cycles at various strain values.

The durability of the stretchable circuits was inspected by applying a radial cyclic test to the printed circuits, where the normalized resistances were recorded against the number of loading cycles. Figure 14 shows the normalized resistances of both the straight line and the horseshoe pattern with a linewidth of 2 mm. Both patterns are printed on the optimal radially pre-stretched sample at 27% strain.

As expected, the normalized resistance increases as the number of loading cycles and the amount of applied strain increase. Below a cyclic strain of 5% the straight line is more stable, with an almost constant normalized resistance compared to the horseshoe pattern. On the other hand, for 10% and 15% cyclic strains the change in the normalized resistance of the horseshoe pattern after 200 cycles is less than that of the straight line. This indicates that the horseshoe pattern sustains larger cyclic strain. It is worth mentioning that the small fluctuations in the normalized resistances, shown in Figure 14, are caused by the instantaneous change in the multimeter readings due to motion artifacts between the multimeter leads and the conductive pattern during the cyclic test. It should be noted that the resistance of both patterns remained well below the kOhm range during the test, as shown in Table 2.

## 5. Conclusions

This study analyzed and compared the effects of axial and radial pre-stretching on the stretchability of inkjet-printed circuits. Moreover, it inspected the effects of the combination of the pre-stretching methodology and the use of horseshoe patterns on the stretchability of these circuits. Results indicate that radially pre-stretched circuits can sustain radial loads better than axially pre-stretched ones. Specifically, a circuit radially pre-stretched by a strain of 27% demonstrated optimal performance under radial loads where the maximum average breakdown strain was almost 19%. This is suitable for applications where stretchable circuits need to be fitted to 2D curvatures, such as the human body.

Additionally, horseshoe patterns, which are commonly used in stretchable circuits to sustain axial loads, were tested under radial loads. Results imply that straight lines printed on radially pre-stretched substrates can sustain radial loads better than horseshoe patterns, even with radial pre-stretching. In particular, a horseshoe pattern with two layers and a linewidth of 1.5 mm sustained up to 13.5% radial strain on average, and a pattern with one layer and a linewidth of 2 mm sustained up to 16% radial strain on average. Therefore, it can be concluded that there is no need to use wavy patterns, which require a meticulous printing process, as straight lines printed on radially pre-stretched substrates perform better under radial loads.

It is worth mentioning that the dimensions of the printed patterns presented here are only used as a proof of concept, as they can be scaled down to develop stretchable circuits in the micro- or nanoscale. Additionally, the outcome of this work is applicable to a broad spectrum of stretchable sensors that can be placed on different parts of the human body, such as pulse oximeters which are used to measure pulse rate and oxygen saturation [16].

## Figures and Tables

**Table 1:** Initial and final resistances and average breakdown strain of horseshoe patterns with and without radial pre-stretching.

Pre-Stretching Strain	0%			27%		
Number of Layers	1		2	1		2
Line Width	1.5 mm	2 mm	1.5 mm	1.5 mm	2 mm	1.5 mm
$R_{i}$ (Ohm)	20	22	9.3	69.1	40	37
$R_{f}$ (Ohm)	98	380	14.5	698	279	84
Average Breakdown Strain (%)	4.5	9	4.5	13	16	13.5

**Table 2:** The initial, maximum, and final resistances of the straight line and the one-layer 2 mm horseshoe pattern during the radial cyclic test.

Pattern	Straight-Line			Horseshoe		
Cyclic Strain	5%	10%	15%	5%	10%	15%
$R_{i}$ (Ohm)	21.96	29.13	38.54	50.26	67	68.9
$R_{\max}$ (Ohm)	90.52	286.43	565.75	270.47	528.36	595.02
$R_{i1000}$ (Ohm)	43.83	219.05	119.13	101	141	200.47
